# Control of STIs and HIV: The male reproductive and sexual health context – The paradigms

**DOI:** 10.4103/2589-0557.69006

**Published:** 2010

**Authors:** Karun Dev Sharma

**Affiliations:** Department of Community Medicine, G.S.L. Medical College, NH 5, Lakshmipuram, Rajahmundry, Andhra Pradesh - 533 296, India

Sir,

The concern about male reproductive and sexual health and, concomitantly, the health of their sexual partners is set in the context of rapid spread of human immunodeficiency virus (HIV) and increasing rate of sexually transmitted infections (STIs) in India. In the context of HIV prevention, a reduction in the prevalence of other STIs can help in decreasing the rate of transmission of HIV.

In the present scenario, men, in general, do not feel comfortable seeking services from family planning clinics and young people, in particular, often feel embarrassed.[1] Men’s reproductive health needs include a wide range of services such as family planning, treatment and prevention of STI/HIV, infertility, sexual problems and others. Men need clinics and staff that provide confidential and non-judgmental care.[2] Also, to increase the male participation in reproductive and sexual health, the primary focus should be on strategies to “encourage and enable men to take responsibility for their sexual and reproductive behavior and their social and familial roles.” The time is now ripe to determine and address men’s sexual health needs if they are expected to participate fully as responsible partners in improving and protecting their own and their partner’s sexual and reproductive health.[3]

Under the Research and Intervention in Sexual Health: Theory to Action (RISHTA) project at the International Institute for Population Sciences, a male health clinic was established in one of the three experimental communities. RISHTA interventions successfully demonstrated the strategies of effective community health education programmes and also the efficacy of engaging Ayurveda, Unani, Siddha, Homeopathy and Allopathic providers in HIV/STI risk reduction among men.[4] PROFAMILIA in Colombia offers men their own all-male clinic located in a different building from the regular clinics, and also has found the number of male clients increasing at its regular clinics when staffs are trained to be sensitive to men’s unique needs.[1] In rural Bangladesh, reproductive health services for men were successfully integrated into formerly female-focused services without compromising the quality of care. The addition of services for men increased utilization of clinical services by both men and women.[5] The Talking about Reproductive and Sexual Health Issues telephone helpline based in New Delhi receives the majority of its calls from young men. On subsequent calls, male clients reported delaying penetrative sex, masturbating instead of visiting a commercial sex worker and adopting other less risky sexual practice.[1]

The choice of the paradigm in a community will depend on the pattern of utilization of health services by males, type of services already existing and the resources available. The exclusive male health clinic model poses a challenge of intensive resource requirement for establishment and maintenance. Being in the era of integrated health programmes, a similar model shall thus be a practicable choice.
